# Extensive experimental investigation on the effect of thermal treatment and lateral pressure on the shear behavior of intact mudstone

**DOI:** 10.1038/s41598-023-33841-5

**Published:** 2023-04-26

**Authors:** Mahmoud Alneasan, Abdel Kareem Alzo’ubi

**Affiliations:** 1 Civil Engineering Department, Tishreen University, Latakia, Syria; 2 Civil Engineering Department, Abu Dhabi University, Al Ain, UAE

**Keywords:** Civil engineering, Solid Earth sciences

## Abstract

The coupling environment of temperature (T) and lateral pressure at great depths promotes intact rocks to shear failure, posing a serious threat to underground engineering. Temperature effect on shear behaviour is of particular importance due to the possible mineralogical alterations in mineral composition, especially in clay-rich rocks such as mudstone that has a great affinity for water. Accordingly, the effect of thermal treatment on the shear behaviour of intact mudstone was investigated, in this study, using the Short Core in Compression (SSC) method. Three temperatures of RT, 250 and 500 °C, and four lateral pressures of 0.0, 0.5, 2.0, and 4.0 MPa were adopted. Numerical and experimental observations showed that the resulting fractures in SCC samples are shear and by increasing the lateral pressure, shear failure is promoted. Compared with other rock types such as granite and sandstone, shear properties in mudstone have only one positive trend with temperature increase up to 500 °C, by increasing T from RT to 500 °C, mode II fracture toughness, peak friction angle, and the cohesion increased by about 15 to 47%, 4.9%, and 47.7%, respectively. The bilinear Mohr–Coulomb failure criterion can be used to model the peak shear strength behaviour of intact mudstone before and after thermal treatment.

## Introduction

The earth’s crust is exposed to many environmental factors that might threaten the safety and stability of geological structures, surface, and underground infrastructures. Due to the change caused by temperature on the mineral composition and degree of cracking induced by thermal stress^1–3^, the temperature is one of the most important factors directly affecting the mechanical behaviour of geological structures including intact rocks and fracture networks. Taking into account the coupling effect of temperature and lateral pressure at greater depths, where the average rate of increase in temperature with depth is 3–5 °C/100 m^4^, intact rocks become more prone to shear failure^5^ which causes a severe threat to deep engineering^6^. Therefore, investigating the temperature-dependent shear behavior of intact rocks is of great importance to analyze the safety and stability of underground engineering structures under the condition of thermo-mechanical (TM) coupling.

Numerous contributions have been made by many scholars to analyze rock behavior under thermal treatment effects. Here we highlight recent works related to the temperature-dependent shear behavior of intact rocks, which is the focus of the current research. Shao et al.^7^ investigated the mechanical behaviour of intact granite under in-situs stress and temperature conditions using cylindrical rock specimens. They concluded a reduction in shear parameters by increasing the temperature, and the conventional Mohr–Coulomb criterion failed to model shear behavior under geothermal reservoir conditions. Zhai et al.^8^ studied shear characteristics of healed joints and intact granite after thermal treatment and showed that the ductile failure characteristics of these joints after high-temperature thermal treatment are greater than that in intact rocks. In addition, they pointed out that dilation angle, shear stiffness, and peak shear strength are positively correlated with temperature up to 400 °C, and negatively correlated with T greater than 400 °C. Chen et al.^6^ investigated the shear behavior of intact cubic specimens of granite under thermo-mechanical coupling and showed that the internal friction angle and cohesion significantly decreased with an increase in the temperature, while the roughness of resulting fracture was directly proportional to temperature increase. Zhu et al.^9^ showed that the cohesion and internal friction angle of intact cylindrical specimens of granite reduces with temperature, decreasing rapidly by 49.39% and 27.51% from 500 to 600 °C respectively. The effect of thermal treatment on the shear behavior of intact sandstone was investigated by Liu et al.^10^. They concluded that the shear strength first increases before 600 °C and decreases after 600 °C, the shear modulus varies slightly before 600 °C and decreases rapidly after 600 °C. Yin et al.^11^ studied the shear mechanical behavior of intact sandstone after thermal treatment at temperatures between 100 and 800 °C. They pointed out that due to decreasing peak shear strength and enhanced ductility for temperatures between 400 and 800 °C, the secant peak shear stiffness declined by 43.79–70.48%.

**Figure 1:**
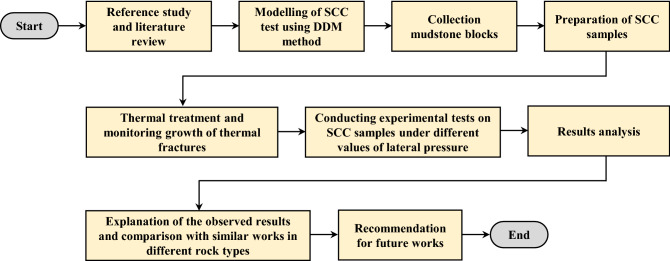
Flow chart visualizing the steps taken to develop this study.

It can be noted that most research related to the effect of temperature on the shear behaviour of intact rocks were conducted on igneous rocks such as granite. While sedimentary rocks such as sandstone, limestone, and mudstone that are closely located above and below oil and gas reservoirs and coal beds^12^ have received less attention. Moreover, clay-rich rocks such as mudstone are widely distributed in the earth's crust, accounting for approximately 30% of the surface rocks^13,14^. These rock types have a great affinity for water due to the formation of a diffuse double layer around the negatively charged clay minerals, this makes the mechanical properties of these rocks unique and more complicated when exposed to temperature effects^15–17^. Some attempts were conducted to investigate the compressive and tensile behaviour of mudstone after thermal treatment^12,16–25^, and concluded that tensile and compressive properties improve to a certain temperature (400 and 600 °C) due to drying of clay minerals during thermal treatment, and then decrease after this limit. On the other hand, the effect of thermal treatment on shear behaviour of intact mudstone needs a comprehensive study and extensive efforts. Therefore, the novelty of this study comparing with the existing literature lies on the coupling effect of temperature and lateral pressure on shear behavior of clay rich rocks in its intact form. Shear behavior of intact mudstone after thermal treatment was investigated following the flowchart shown in Fig. 1 which visualizes the steps taken to develop this study.

## Methods

The effect of thermal treatment on the shear behaviour of intact mudstone was investigated by using the Short Core in Compression (SCC) test. Two methods were adopted to achieve the goal of the current research, numerical and experimental methods. The numerical method was used to simulate the SCC sample to obtain stress fields and mode II stress intensity factor (K_II_) under uniaxial and lateral stress states, and to judge the type of the resulting fracture, whether is it a tensile or shear fracture. While the experimental method was used to obtain real results for the effect of thermal treatment on shear characteristics of intact mudstone.

### Numerical simulation of SCC test

**Figure 2:**
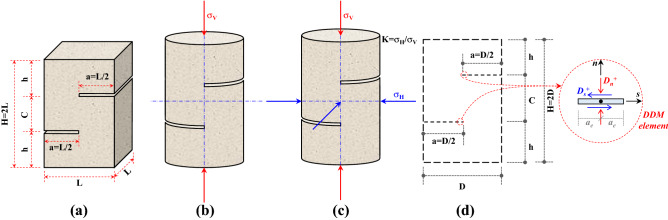
Short core in compression test, (**a**) short beam in compression (SBC), (**b**) short core in compression under uniaxial stress state, (**c**) short core in compression under confined stress state, (**d**) modelling of SCC samples using displacement discontinuity method.

Short core in compression samples were used in this study to investigate the effect of temperature and lateral pressure on the shear properties of intact mudstone. These samples were utilized here to simulate a continuous and intact rock bridge under shear forces, such that the new fracture grows in the rock bridge under pure shear stress. This bridge is subjected to normal and shear stresses and reflects the properties of the intact material. Short core in compression test is generally used to investigate shear fractures that grow between tips of two horizontal notches. In the beginning, Watkins and Liu^26^ developed the Short Beam in Compression (SBC) test by using squared pillars shown in Fig. 2a. Since squared pillars require extensive machining work compared to cylindrical specimens that can be easily prepared by extracting a core from a rock block, Jung et al.^27^ suggested Short Core in Compression (SCC) test shown in Fig. 2b. The advantage of these samples is the possibility of applying lateral pressure by using Hoek cell to investigate shear fractures under different values of horizontal stress σ_H_ (Fig. 2c). To obtain stress fields over the entire rock sample and stress intensity factor K_II_ at the notch tip, the SCC test was numerically modeled using the Displacement Discontinuity Method (DDM) which is a branch of the Boundary Element Method (Fig. 2d). In this method, boundaries of the rock sample are divided into N DDM elements, and each element is subjected to two types of boundary conditions that include normal stress σ_n_, shear stress σ_s_, normal displacement u_n,_ and/or shear displacement u_s_. A system of algebraic equations (N × N) is formed between boundary conditions and displacement discontinuities (D_n_ and D_s_) and by solving this system, D_n_ and D_s_ are obtained for each element. Where D_n_ and D_s_ are normal and shear displacement discontinuities, respectively. Normal displacement discontinuity D_n_ can be defined as the difference in normal displacement u_n_ between the two sides of the DDM element. A positive D_n_ means that the DDM element is under compression and the element surfaces approach each other, and vice versa for negative D_n_. Shear displacement discontinuity D_s_ can be defined as the difference in shear displacement u_s_ between the two sides of the segment, and the positive value of D_s_ means that the upper surface moves to the left relative to the lower surface (left lateral slip). Finally, stress components, displacements, and stress intensity factors at the midpoint of each element are calculated as a function of D_n_ and D_s_^28^. By using the DDM method, a numerical code was developed in our previous works to predict fracture path, stress fields, and stress intensity factors in different rock samples^18–20,29^. This code was extended in this study to model the SCC test under uniaxial and confined stress states. Based on the simulation that is shown in Fig. 7 and the experiments conducted by previous researchers on SCC specimens^27,30,31^, the maximum shear stress concentrates at the tips of the two notches and shear fracture initiates and begins to grow perpendicular to these notches.

**Figure 3:**
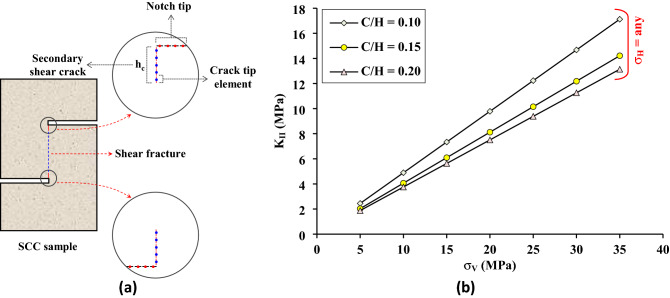
Stress intensity factor in SCC specimens, (**a**) modeling of the secondary shear crack in SCC specimen using DDM, (**b**) mode II stress intensity factor versus σ_V_ and C/H ratio for any value of lateral stress σ_H_.

Based on the fracture mechanics theory in linear elastic fracture mechanics^32,33^, determining the stress intensity factor K_II_ involves modelling the development of a secondary shear crack from the notch tip, which is the initiation point for the growth of the shear fracture (Fig. 3a). In other rock specimens that are used to generate shear fractures under both mode II loading and mode II initiation such as shear box and punch-through shear (PTS) specimens, a secondary shear crack is not necessary for simulation analysis to obtain the K_II_ because the new crack plane is along the notch plane. While in the SCC specimen, the plane of the new shear crack is perpendicular to the notch plane. Yao et al.^31^ pointed out that the maximum shear stress concentrates along the vertical bridge between two notch tips and it is maximized at these tips, the fracture initiates at lower and upper notch tips, and secondary shear cracks are required to calculate K_II_. Moreover, by using the digital image correlation technique on SCC samples of granite, Zhang et al.^34^ found that new cracks first initiate from two notch tips and then propagate toward the central area in the expected shear fracture band. To apply the traditional fracture mechanics theory to the SCC specimen, the secondary shear crack is necessary for accurately determining the stress intensity factor in the fracture process, and the K_IIC_ can be further determined precisely when a crack tip exists along the shear bridge between tips of the two horizontal notches^35,36^. Accordingly, a secondary shear crack with length h_c_ was modeled in this study and divided into N_c_ elements, the final element was treated as a crack tip element. Displacement discontinuities (D_n_^Tip^ and D_s_^Tip^) were calculated at the crack tip element and the mode II stress intensity factor K_II_ was obtained as a function of the shear displacement discontinuity (D_s_^Tip^) using the following equation of Shou and Crouch^37^:

1 $${\mathrm{K}}_{\mathrm{II}}=\frac{\mathrm{G}}{4\left(1-\upnu \right)}\sqrt{\frac{2\uppi }{{\mathrm{a}}_{\mathrm{e}}}}{\mathrm{D}}_{\mathrm{s}}^{\mathrm{Tip}},$$

where G and ν are shear modulus and Poisson’s ratio, respectively. D_s_^Tip^ is the shear displacement discontinuity at the crack tip element. a_e_ is half the length of the crack tip element. Figure 3b shows mode II stress intensity factor K_II_ as a function of σ_V_, σ_H,_ and C/H ratio. Figure 3b shows that the K_II_ is not affected by the lateral stress σ_H_, this is because K_II_ is mainly a function of the shear stress at the secondary shear crack tip (D_s_^Tip^ = *f*(σ_xy_^Tip^)), and as observed in the numerical model in Fig. 7, when the lateral stress σ_H_ is increased, the shear stress along the bridge between two notches remains constant. In addition, Fig. 3b shows that the K_II_ increases with increasing the vertical stress σ_V_, and it is inversely proportional to the C/H ratio. These results are in complete agreement with the numerical observations of Xu et al.^30^ and Yao et al.^31^ obtained using the FEM method.

### Experimental procedure

**Figure 4:**
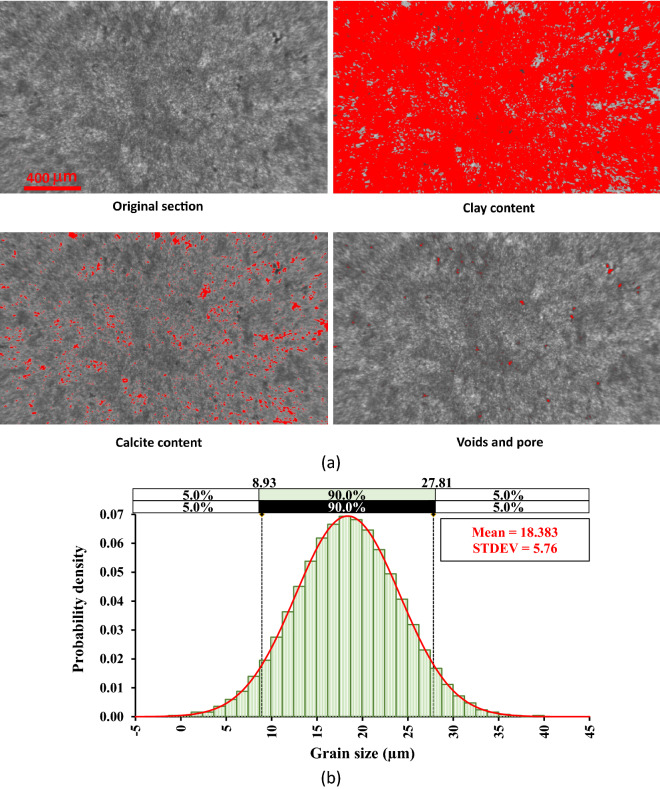
(**a**) Mineralogical composition, (**b**) grain size distribution.

The objective of this research is to examine how temperature and lateral pressure impact shear fractures in clay-rich rocks. Therefore, it was essential to choose a rock type that is abundant in clay to meet the aim of this study. As a result, mudstone rock was chosen due to its mineral composition and grain size distribution as depicted in Fig. 4a. The gray and white shades in the original section of Fig. 4a symbolize clay and calcite, respectively. While the black color represents voids and pores. The proportion of these components was determined through color analysis using ImageJ software. The mudstone sample used in this study is composed of 92.75% clay, 7% calcite, and 0.25% voids and pores. Moreover, the grain size distribution shown in Fig. 4b was determined using ImageJ and @Risk software. It is worth mentioning that 90% of the mudstone sample used in this research is made up of grains with sizes ranging from 8.93 to 27.81 μm. ImageJ is a public-domain Java image processing program inspired by NIH Image. This software can be used to measure grain sizes due to the following features; ImageJ can calculate area and pixel value statistics of user-defined selections, measure distances and angles, create density histograms and line profile plots, supports standard image processing functions such as contrast manipulation, sharpening, smoothing, edge detection and median filtering. @RISK software is an add-in tool for Microsoft Excel that helps to make better decisions through risk modeling and analysis. This software has 33 distribution functions that can be used to determine grain size distribution.

The physical and mechanical properties of the mudstone tested in this study are summarized in Table 1. These properties were measured in this study according to established standards in rock mechanics (ISRM)^38^. As shown in this table, the mudstone used in this study is characterized by low porosity of 0.664%, relatively low tensile strength of 3.2 MPa, and high compressive strength of 109 MPa.

**Table 1:** Physical and mechanical properties of the tested mudstone in this study.

Property type	Physical properties					Mechanical properties						
Property	Density	Porosity	Void ratio	Moisture ratio	Saturation ratio	UCS	BTS	P-wave	S-wave	R-wave	Young’s modulus	Poisson’s ratio
Symbol	ρ	n	e	w	S_r_	σ_c_	σ_t_	C_p_	C_s_	C_r_	E_d_	ν_d_
Unit	gr/Cm^3^	%	%	%	%	MPa	MPa	m/s	m/s	m/s	GPa	–
Magnitude	2.66	0.664	0.669	0.15	60	109	3.2	4575	2673	2448	43.45	0.24

### Sample preparation

**Figure 5:**
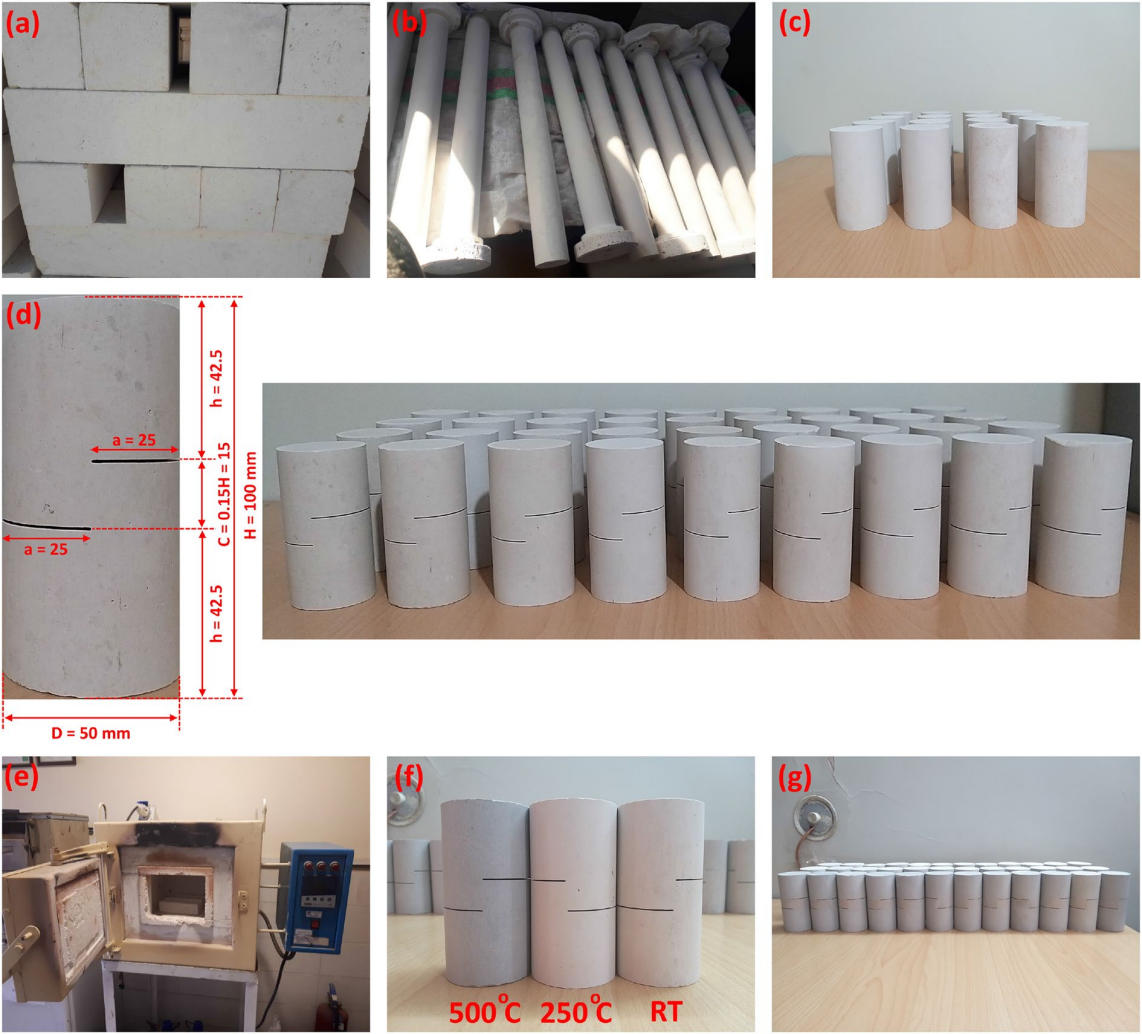
Stages of preparation of rock samples, (**a**) search for rock blocks, (**b**,**c**) cylindrical sample preparation, (**d**) SCC samples after making two artificial notches, (**e**) furnace used for thermal treatment, (**f**,**g**) SCC samples after thermal treatment.

Figure 5 shows the stages of preparation of the rock samples. To ensure homogeneity between samples, all samples were prepared from the same rock block. Figure 5a shows a group of prismatic samples of the mudstone taken from a single block. Then the prismatic samples were transformed into cylindrical samples with a diameter of 5 cm through a process of rotating, carving, and polishing (Fig. 5b). The resulting samples were divided into smaller specimens with a height of 10 cm so that the ratio of height to diameter is 10/5 = 2 (Fig. 5c). The cylindrical rock specimens in Fig. 5c were prepared according to ASTM D7012, where the length-to-diameter ratio (H/D = 2), and the cylindrical surfaces are prepared to be flat and smooth. In particular, the sample’s ends must be leveled within a 0.02 mm tolerance and they should not depart from perpendicularity by more than 0.06 degrees. To obtain SCC samples, two parallel notches were made in each cylindrical specimen according to the dimensions shown in Fig. 5d. These measurements were chosen as a result of the numerical analysis outlined in this study, and depending on previous investigations of Jung et al.^27^ and Xu et al.^30^.

The SCC samples were subjected to thermal treatment using the furnace depicted in Fig. 5e. This furnace has a maximum temperature of 1200 °C and a heating rate of 4.2 °C per minute. The SCC samples were divided into three groups: the first group was not thermally treated and was tested at room temperature (RT). The second and third groups were heated in the furnace for 24 h at temperatures of 250 and 500 °C, respectively. As will be explained later, the maximum temperature of 500 °C was adopted here as the maximum temperature below which no thermal fractures were observed on the mudstone surface. Thermal fractures greatly affect the continuity and properties of SCC samples, which makes these samples heterogeneous and the obtained results more dispersed. Once the 24 h were over, the furnace was turned off and the temperature was allowed to decrease back to room temperature. To prevent thermal shock, the samples were heated and cooled gradually. As shown in Fig. 5f and Fig. 5g, the SCC samples’ color changed depending on the thermal treatment temperature. The original beige color of the mudstone samples at RT changed to a light creamy color after treatment at 250 °C. While after 24 h in a furnace at a temperature of 500 °C, the SCC samples acquire a gray color (cement color). As will be shown later, these color changes are due to the formation/decomposition of calcium compounds and clay minerals which are the main components of the mudstone.

### Experimental apparatus

**Figure 6:**
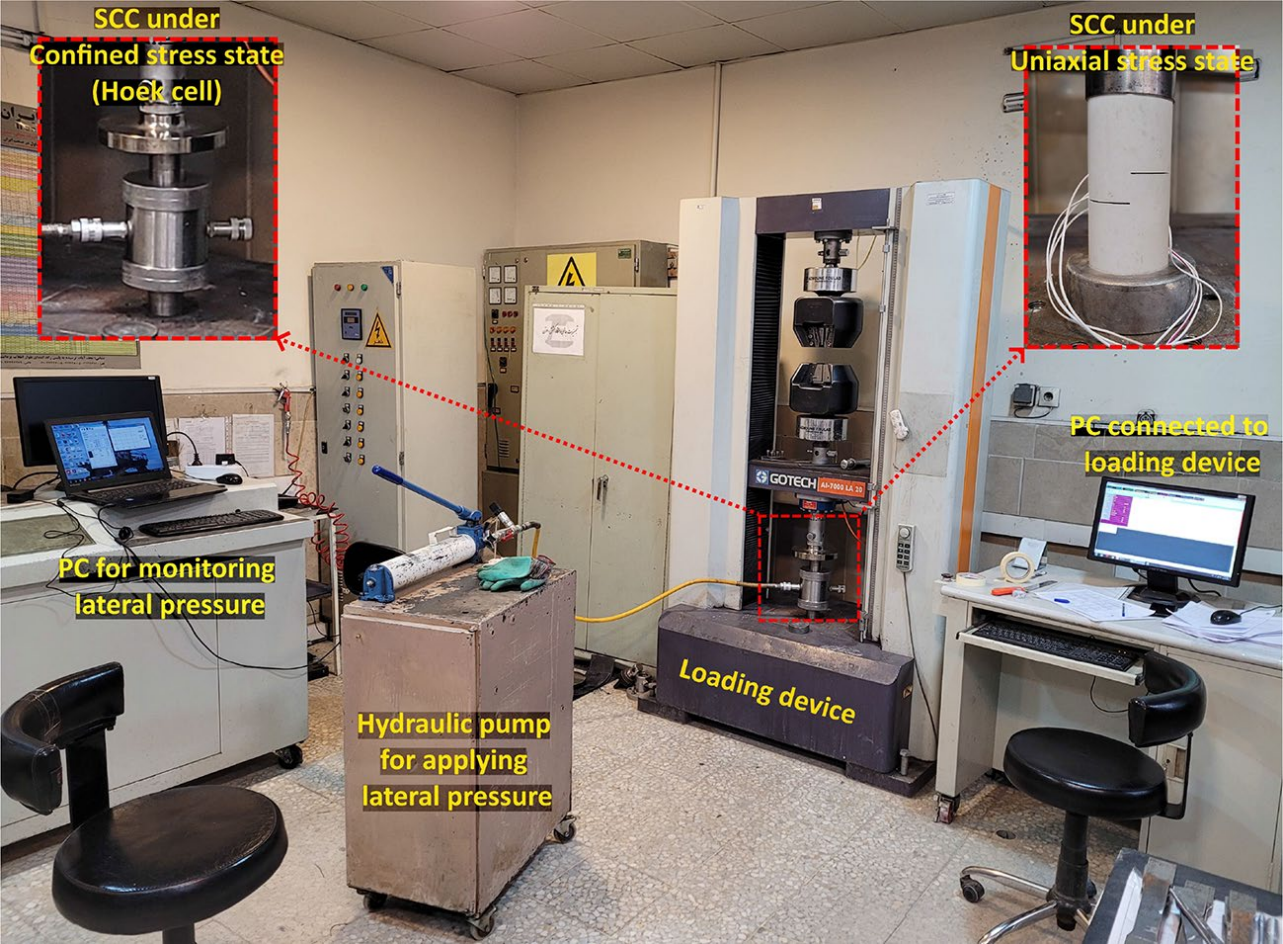
Loading device and lateral pressure unit used in this study to test SCC samples under uniaxial and confined stress states.

The SCC samples were tested under uniaxial and confined stress states using a servo-hydraulic machine shown in Fig. 6. The machine used is a GOTECH AI-7000 LA 20 with a capacity of 20 T and a vertical displacement speed range of 0.0001 to 250 mm/min. All SCC samples were tested under a vertical speed of 1 mm/min so that the resulting strain rate was 1.7 × 10^–4^ S^−1^. A hydraulic pump with a Hoek cell was used to apply three levels of lateral pressure, 0.5, 2, and 4 MPa. The lateral pressure was maintained constant until the point of fracture and it was monitored and recorded via a PC connected to the hydraulic pump.

## Results and discussion

### Shear and maximum principal stress fields predicted by numerical method

**Figure 7:**
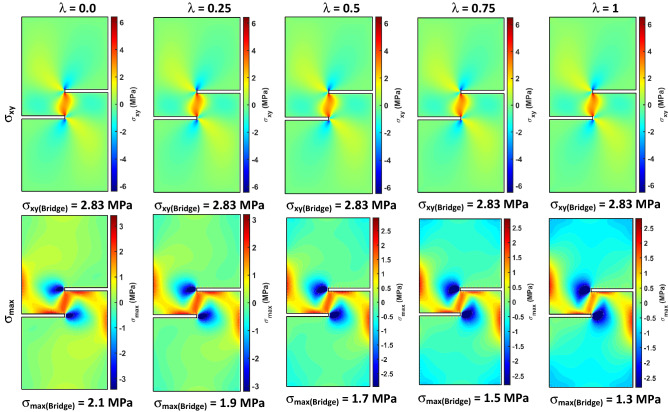
Shear stress σ_xy_ and maximum principal stress σ_max_ as a function of stress ratio λ = σ_H_/σ_V_ in SCC specimens for (H = 100 mm, D = 50, C = 15, a = 25).

Distribution of shear stress σ_xy_ and maximum principal stress σ_max_ over the entire SCC sample as a function of stress ratio λ = σ_H_/σ_V_ is shown in Fig. 7. In numerical calculations used to obtain stress fields in this figure, σ_V_ was constant equal to 1 MPa, but σ_H_ was changed from 0.0 to 0.25, 0.5, 0.75 and 1.0 MPa to obtain stress ratio λ from 0.0 to 1.0. Figure 7 shows that the shear stress σ_xy_ concentrates along the bridge between tips of the two horizontal notches. Note that, the distribution and magnitude of σ_xy_ is not affected by increasing stress ratio λ. Moreover, the maximum value of shear stress along the bridge between the two notches σ_xy(Bridge)_ is constant and equals 2.83 MPa for λ from zero (uniaxial case) to λ = 1 (hydrostatic case). The maximum principal stress σ_max_ was monitored and it can be noted that this stress forms an inclined bridge between the tips of the two horizontal notches, and it is positive indicating tensile stress development along this bridge, which may result in the growth of the tensile fracture instead of the shear fracture between the two notches. Therefore, the resulting fracture must be evaluated to determine if it is a shear fracture due to σ_xy_ or tensile fracture due to σ_max_. In general, shear fractures are vertical between the tips of the two notches, while tensile fractures are inclined to the right relative to the vertical long axis of the SCC sample. In addition, the surface of shear fractures is smoother and flatter than the tensile fractures, especially when the lateral pressure σ_H_ is applied^39^. Additionally, Fig. 7 shows that the distribution (width of the stress zone) and magnitude of σ_max_ decreases with increasing the stress ratio λ. The maximum value of σ_max_ along the bridge between tips of the two notches σ_max(Bridge)_ decreases by about 38% when the λ increases from zero to one. Therefore, by exerting lateral pressure on SCC samples, the impact of tensile stress is reduced and the growth of shear fractures is promoted.

### Displacement load curves

**Figure 8:**
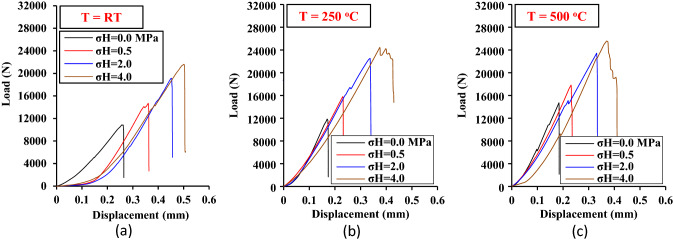
Displacement-load curves of the tested SCC samples at three temperatures of (**a**) RT, (**b**) 250 and (**c**) 500 °C.

The SCC samples were tested under three temperatures (RT ≈ 25, 250, and 500 °C) and four levels of lateral pressure (0.0, 0.5, 2, and 4 MPa) with three tests conducted for each condition, resulting in a total of 36 tests. The load–displacement curves under RT, 250 and 500 °C (for the four lateral pressures) are shown in Fig. 8a–c, respectively. To simplify the presentation and comparison of results, one load–displacement curve was presented for every three replicate tests (one curve per lateral pressure). Figure 8 illustrates that as the temperature and lateral pressure increase, the peak (fracture load) and the area under the load–displacement curve also increase. A higher peak indicates greater resistance to fracture growth. However, a larger area under the curves indicates a greater amount of strain energy stored in the rock sample before the fracture instant, which greatly impacts the speed of the fracture growth between the tips of the two horizontal notches.

### Fracture toughness

**Figure 9:**
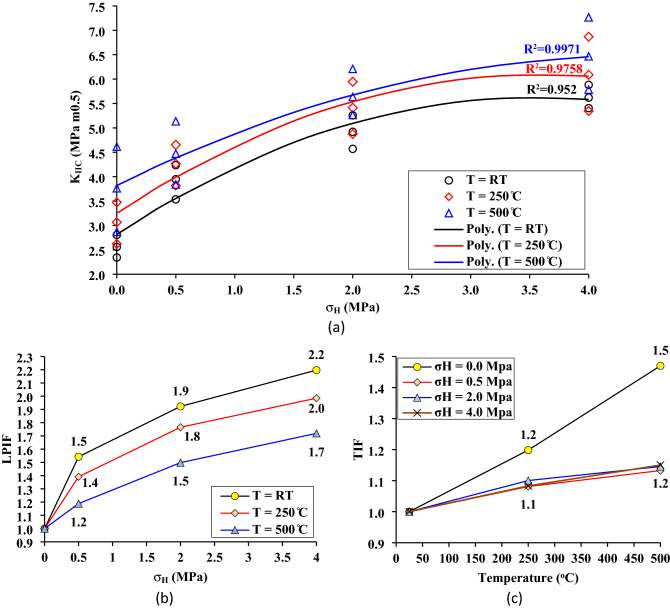
Fracture toughness, (**a**) fracture toughness as a function of the temperature and lateral pressure, (**b**) lateral pressure increasing factor (LPIF), (**c**) temperature increasing factor (TIF).

Fracture toughness, measured by the stress intensity factor at the point of fracture, is a key indicator of a rock’s fracture resistance to grow^40^. In other words, fracture toughness K_IIC_ corresponds to the stress intensity factor K_II_ (Eq. (1)) for P = P_cr_, where P_cr_ is the peak of the load–displacement curve (Fig. 8). Thus, the numerical code developed for this study was used to calculate the fracture toughness of the SCC samples by identifying P = P_cr_, and the results are presented as a function of the temperature and lateral pressure in Fig. 9a. This figure illustrates how temperature and lateral pressure increases the fracture toughness. As will be demonstrated later, the drying of clay minerals after thermal treatment enhances the mechanical properties of rocks rich in clay. Additionally, the lateral pressure σ_H_ acting perpendicular to the shear fracture increases the shear strength between the newly fractured surfaces, causing the K_IIC_ to increase with the increase of σ_H_.

To demonstrate the impact of temperature and lateral pressure separately on the fracture toughness, two parameters are introduced in this study; Lateral Pressure Increasing Factor (LPIF) and Temperature Increasing factor (TIF). The LPIF which is the K_IIC_ under σ_H_ normalized by K_IIC_ under a uniaxial stress state is shown in Fig. 9b, and the TIF which is the K_IIC_ after thermal treatment normalized by K_IIC_ at room temperature is shown in Fig. 9c. Figure 9b illustrates that as σ_H_ increases, the LPIF also increases and that as the temperature of thermal treatment increases from RT to 250 and 500 °C, the impact of lateral pressure on fracture toughness decreases. Therefore, it can be inferred that mudstone is more affected by lateral pressure at normal temperatures than at elevated temperatures. The effect of thermal treatment on K_IIC_ is shown in Fig. 9c, it can be observed that under a uniaxial stress state (σ_H_ = 0.0 MPa), the K_IIC_ is affected by thermal treatment more than that under a confined stress state (σ_H_ = 0.5, 2 and 4 MPa). Comparing LPIF in Fig. 9b and TIF in Fig. 9c, it can be deduced that the mudstone is more affected by lateral pressure than temperature. The LPIF varies between 1.0 and 2.2, while TIF changes between 1.0 and 1.5.

### Peak shear strength behavior

**Figure 10:**
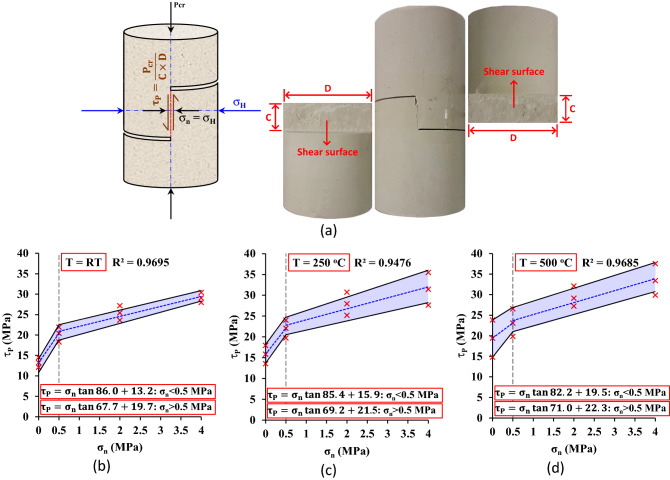
(**a**) Schematic diagram of the SCC sample containing rock bridge with a tested sample of mudstone showing shear surfaces, (**b**–**d**) peak shear strength/normal stress behavior of intact mudstone under RT, 250 and 500 °C, respectively.

In this study, the shear strength behavior of an intact bridge in mudstone was investigated using SCC samples under the effect of temperature and lateral pressure. A schematic diagram of the SCC sample containing a rock bridge tested sample, and shear surfaces is shown in Fig. 10a. As shown in the figure, the intact rock bridge is subjected to normal stress σ_n_ = σ_H_ and shear stress τ = P/(C × D). At the moment of fracture, the peak shear strength can be calculated by τ_p_ = P_cr_/(C × D). The peak shear strength τ_p_ versus normal stress σ_n_ under RT, 250 and 500 °C is shown in Fig. 10b–d respectively. It can be noted that the peak shear strength behavior of a continuous bridge in mudstone (τ_p_ = σ_n_ tan φ_p_ + C_p_) follows the bilinear Mohr–Coulomb failure criterion between shear stress τ and normal stress σ_n_ of intact materials (τ = σ_n_ tan φ + C). The first linear equation is for σ_n_ between 0.0 and 0.5 MPa, while the second one is for σ_n_ between 0.5 and 4 MPa. An important point should be distinguished between the peak shear strength behavior shown in this study and the Mohr–Coulomb relationship. According to the Coulomb behavior in a jointed rock mass, the increment ratio of shear stress to normal stress Δτ/Δσ_n_ represents the friction coefficient f = tan φ between joint surfaces. This coefficient usually ranges from zero (f = 0.0, φ = 0.0°) for perfectly smooth joints to one (f = 1.0, φ = 45°) for completely rough joints. Our results indicated that the increment ratio of peak shear strength to normal stress Δτ_p_/Δσ_n_ for σ_n_ > 0.5 MPa changes between (f_p_ = 2.43, φ_p_ = 67.7°) for T = RT and (f_p_ = 2.9, φ_p_ = 71.0°) for T = 500 °C. The friction coefficient (f) or internal friction angle (φ) of continuous rock joints is determined by the slipping between two separated joint surfaces, while in intact rock, the peak friction angle φ_p_ is a result of shearing intact material and can be greater than 45°, as in this study. Yang and Kulatilake^41^ showed that the peak friction angle φ_p_ of granite samples changes from 56.5° for joint persistency K = 0.75 to 66.7° for K = 0.25 ( K is the ratio of joint length to the bridge length). Krsmanović and Langof^42^ and Krsmanovic and Popovic^43^ reported a high peak friction angle between 70° and 80° according to the test results of the limestone. Moreover, Barton^44^ showed in table II in his study peak shear friction angle between 66° and 80° measured on joints during tests at low normal stress.

In this study, it can be observed that increasing the temperature from room temperature (RT) to 250 and 500 °C increases the cohesion C_p_ of intact mudstone under zero normal stress (σ_n_ = 0.0 MPa) from 13.2 to 15.9 and 19.5 MPa, respectively. The peak friction angle (φ_p_) for σ_n_ > 0.5 MPa also increases from 67.7° to 69.2° and 71.0°, respectively. Thus, thermal treatment at higher temperatures improves the mechanical shear properties (such as φ_p_ and C_p_) by 2.2% and 20.5% at 250 °C and by 4.9% and 47.7% at 500 °C. Additionally, when increasing the normal stress σ_n_ from 0.5 to 4 MPa, the peak shear strength τ_p_ increases by 42.4% at T = RT, 42.7% at 250 oC, and 44.6% at 500 °C.

### Numerical and experimental comparison

**Figure 11:**
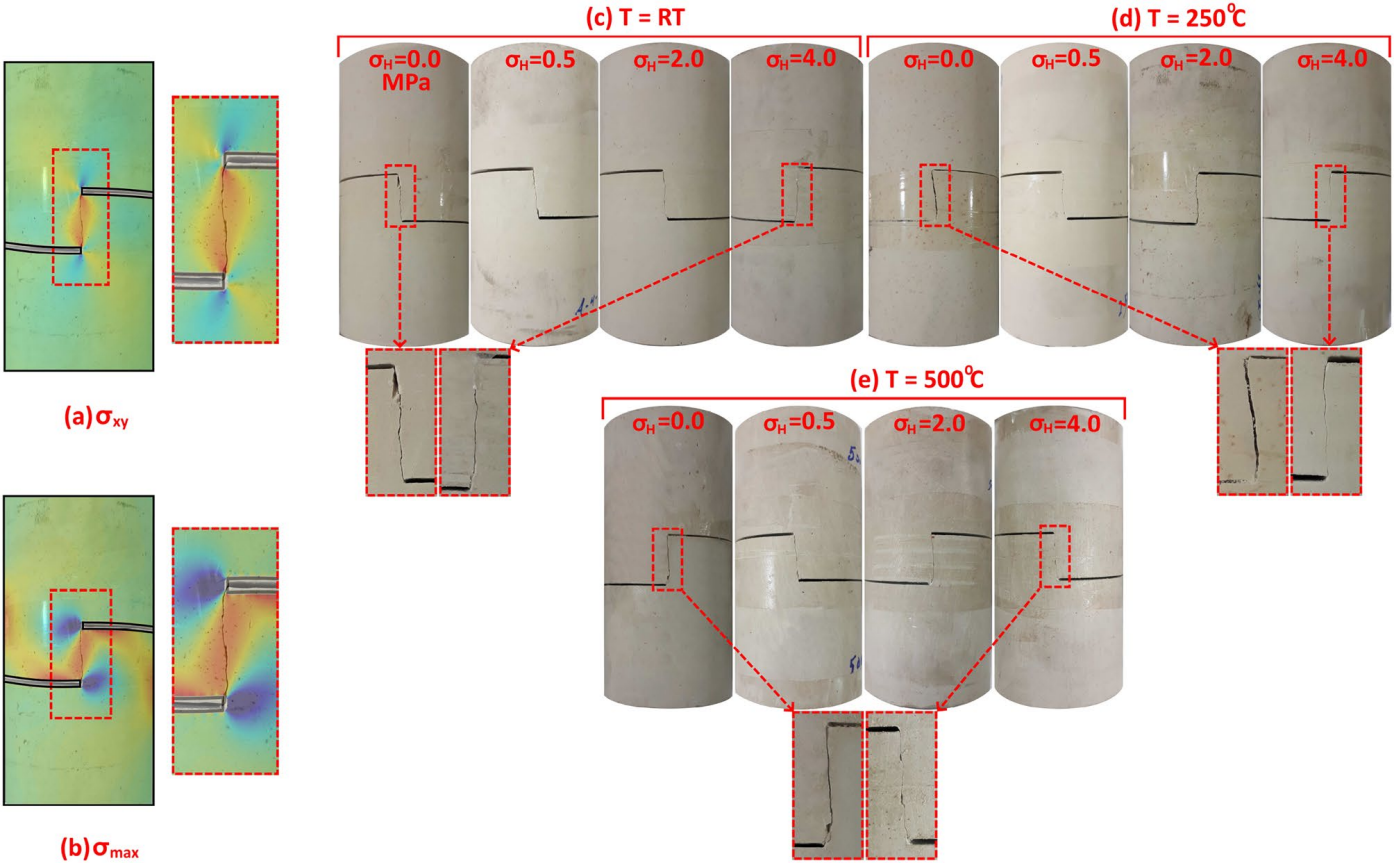
Comparison between numerical results and experimental observations.

A comparison between numerical stress fields predicted in SCC samples and experimental observation for the developed fracture between the tips of the two horizontal notches is shown in Fig. 11. To compare the experimental work and the numerical model, the experimental fracture is superimposed onto the numerical model as shown in Fig. 11a, note that the resulting fracture is a vertical one between the tips of the two notches, which agrees to a large extent with the shear stress develpoed in the numerical model. Moreover, the formed fracture is not inclined such as the maximum principal stress bridge shown in Fig. 11b. Therefore, the resulting rupture surface is a shear fracture. In addition, the resulting fracture was examined for all SCC samples after the test under four lateral pressures and three temperatures by comparing it with numerical stress fileds and it was observed that all induced fractures are shear fractures. Hence the importance of numerical modeling for evaluating induced fractures in rocks. Induced shear fractures in SCC samples at temperatures of RT, 250 and 500 °C are shown in Fig. 11c, Fig. 11d, and Fig. 11e, respectively. Comparing shear fractures induced under the uniaxial case (σ_H_ = 0.0 MPa) and those induced under σ_H_ = 4.0 MPa at three temperatures, it can be seen that the latter are straighter and more healed. This observation agrees also with the numerical result that shows the effect of lateral pressure on induced fracture, it was previously concluded that by increasing the lateral pressure, the impact of tensile stress is reduced and the growth of shear fractures is promoted (See Fig. 7).

### Explanation of the observed results

**Figure 12:**
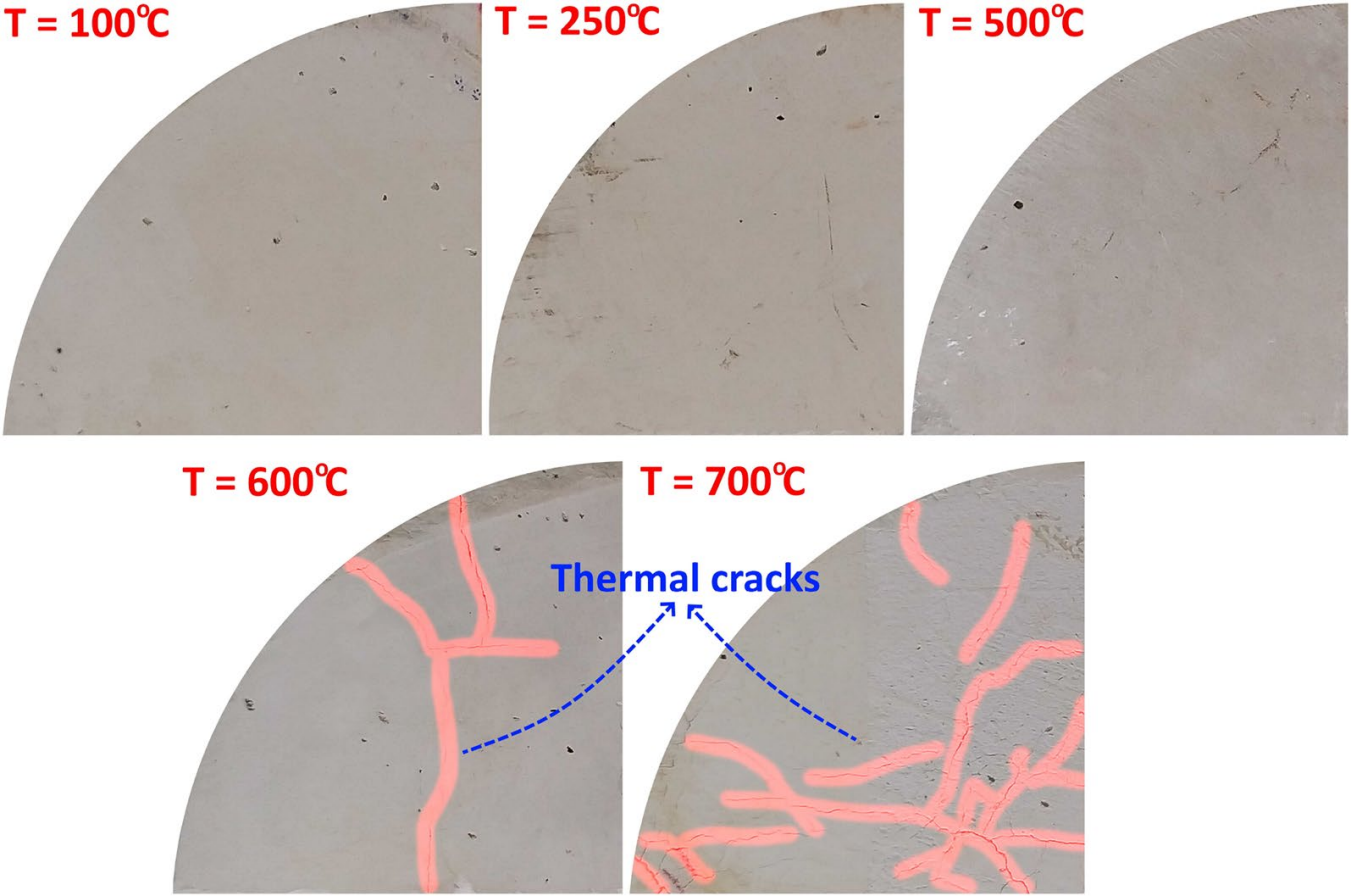
Monitor the growth of thermal cracks after thermal treatment at temperatures of 100, 250, 500, 600 and 700 °C.

This study suggests that thermal treatment improves the mechanical behavior of shear fractures in SCC specimens. The main mechanical properties that were evaluated are the stress intensity factor at the notch tip K_IIC_, peak shear strength τ_p_, peak friction angle φ_p_, and cohesion C_p_. All of these parameters show an improvement with an increase in temperature from RT to 500 °C. As illustrated in Fig. 12, the highest temperature examined for the effect of thermal treatment on the shear properties of SCC samples is 500 °C. This is because at this temperature no thermal cracks were detected on the samples’ surface. It is worth noting that thermal cracks were observed after thermal treatment at a temperature of 600 °C or greater. To explain the observed shear behavior of the mudstone used in this study, a summary of experimental results for the effect of thermal treatment on the shear behaviour of intact rocks is shown in Table 2. Most research in this table focused on the shear behavior of intact granite and showed different regimes for the effect of thermal treatment on shear parameters. Shao et al.^7^ showed that the φ_P_ is slightly affected by temperature increase, it changes between 52° and 55° for a temperature range of RT to 300 °C, while the cohesion C_P_ shows two regimes, increasing by about 23% for T between RT and 100 °C, and then decreasing by about 15% for T from 100 to 300 °C. Zhai et al.^8^ indicated two obvious regimes for temperature effect on shear strength of healed joints and intact granite, the τ_P_ under normal stress ranging from 1 to 9 MPa improved with temperature increase from 25 to 400 °C, and then it decreased for T between 400 and 750 °C. Chen et al.^6^ and Zhu et al.^9^ observed only one negative trend for the effect of thermal treatment on the shear strength properties of Weihai granite and Nanan granite, respectively.

**Table 2:** A summary of experimental results for the effect of thermal treatment on the shear behaviour of intact rocks.

Rock type	Temperature (T, °C)	Observations	Reference
Australian Strathbogie granite	RT, 100, 200 and 300	φ_P_ is constant between RT and 100 °C (φ_P_ ≈ 52°), then slightly increased from 100 to 200 °C (52° to 55°), and then it is constant for T between 200 and 300 °C (≈ 55°)	Shao et al.^7^
		C_P_ increased from 22 to 27 MPa when T increased from RT to 100 MPa, then it decreased from 27 to about 23 MPa for T between 100 and 300 °C	
		The Mohr–Coulomb criterion is not applicable to model shear behaviour	
		No thermal microcracks were observed in granite samples up to 200 °C	
		At higher temperatures,400 °C, some intergranular and intragranular cracks were observed between and in quartz-feldspar minerals	
Hengyang granite	25, 200, 400, 600 and 750	The shear strength of healed joints is highly greater than that of clean joints and slightly lower than that of intact rocks	Zhai et al.^8^
		Compared with intact rocks, the ductile failure characteristics of healed joints are much greater after thermal treatment	
		τ_P_ of healed joints and intact granite increase for T between 25 and 400 °C, and then decrease from 400 to 750 °C	
		Shear stiffness of intact granite and heled joints is negatively correlated to T	
Weihai granite	25, 100, 200 and 300	The peak shear strength is negatively correlated to the temperature	Chen et al.^6^
		When T increased from 25 to 300 °C, φ_P_ decreased by about 31.2% from 48° to 33°, and C_P_ decreased by about 11.7% from 27 to 24 MPa	
		For T from 25 to 300 °C, shear stiffness decreased by about 26%, 27%, and 22% under σ_n_ = 20, 30, and 40 MPa, respectively	
		The roughness increased with temperature due to the increase in complexity of intragranular and transgranular fracturing paths	
Nanan granite	RT, 200, 300, 400, 500 and 600	With increasing T from RT to 600 °C, φ_p_ decreased by about 27% from 37° to 27°, and C_P_ decreased by about 49% from 43 to 22 MPa	Zhu et al.^9^
Neijiang red sandstone	25, 200, 400, 600 and 800	Red sandstone behaves in a plastic manner under high treatment temperatures	Liu et al.^10^
		The shear modulus varies slightly between 25 and 600 °C and then rabidly decreases after 600 °C	
		The peak shear strength increased for T from 25 to 600 °C, and then decreased for T > 600 °C	
		The position of crack initiation varies with the treatment temperature	
		φ_P_ has three regimes with temperature increase, decreasing from 41.4° to 38.4° for T between 25 and 200 °C, increasing from 38.4° to 40.5° for temperature range 200 to 600 °C, a slight decrease from 40.5 to 39 for T between 600 and 800 °C	
		C_P_ has two regimes with temperature increase, increasing from 12.7 to 16.7 MPa for T between 25 and 200 °C, decreasing from 16.7 to 15.7 for T from 200 to 600 °C, and decreasing from 15.7 to 13.3 MPa for T between 600 and 800 °C	
Rizhao yellow sandstone	25, 100, 200, 300, 400, 500, 600, 700 and 800	The peak and residual shear strength display an exponential variation with temperature, a slight increase with T, and then a dramatic decrease, achieving a threshold temperature of 400 °C	Yin et al.^11^
		The secant peak shear stiffness declined by 43.79–70.48% due to decreasing peak shear strength and enhanced ductility for temperatures between 400 and 800 °C	
		For T from 25 to 200 °C, the φ_P_ increased from 42.68° to 44.92°, and then decreased to 30.57° for T = 800 °C	

In addition to the works conducted on intact granite, Table 2 shows two works on the effect of thermal treatment on the shear behavior of sedimentary rocks such as sandstone. These results are consistent in terms of the effect of temperature on the peak shear strength. Liu et al.^10^ observed two regimes for T–τ_P_ relationship in Neijiang red sandstone, the τ_P_ increased for T from 25 to 600 °C, and then decreased for T > 600 °C. In addition, Yin et al.^11^ pointed out that the τ_P_ of Rizhao yellow sandstone has a threshold at T = 400 °C and then dramatically decreased after 400 °C. Regarding our results presented previously in Fig. 10, τ_P_ and C_P_ show only one positive trend with temperature increase, while φ_P_ is inversely correlated to the temperature under normal pressure < 0.5 MPa, and positively correlated to the temperature under normal pressure > 0.5.

The temperature-dependent shear behavior of different rock types is controlled by two main mechanisms, physical and chemical mechanisms^45,46^. The physical mechanism includes the growth of thermal fractures due to the difference in the thermal expansion coefficient of constituent minerals and the evaporation of absorbed water. Thermal expansion of constituent minerals can play a positive or negative role depending on the thermal treatment temperature. For rocks that showed two regimes for the effect of temperature on peak shear strength, increasing shear strength until reaching a threshold at a certain temperature (T_Th_) and then a gradual or rapid decrease. Before T_Th_, positive regime, the thermal expansion of different minerals can cause the reduction of pore volumes and the distance between the interfaces of individual minerals, which increases the contact, bond strength, and mutual attraction between them^47^, which eventually leads to an increase in peak shear strength of intact material. With increasing temperature after the T_Th_, fracture occurs between constituent minerals that show different thermal conductivities and thermo-elastic moduli, resulting in a decrease in peak shear strength^48,49^. For rocks that showed one regime for the effect of temperature on peak shear strength such as in Nana granite, Zhu et al.^9^ explained the observed results using optical microscopy that intergranular microcracks were observed after thermal treatment at 200 °C, and transgranular macrocracks began to appear at 300 °C, and by increasing the temperature, additional thermal microcracks were observed in the rock samples.

The chemical mechanism that controls the temperature-dependent shear behavior indicates the removal of water from rock structure and transformation in crystal type, such as the reaction between biotite and oxygen at above 400 °C^50^, and α to β quartz transition at 573 °C that causes a linear expansion of 0.45% of the quartz^51^. For the mudstone used in this study, the enhancement of mechanical properties as the temperature increases is a result of its high clay mineral content (92.75%) which has a high affinity to water. Towhata et al.^52^ investigated the volume change of clays induced by heating and showed that when the temperature rises, material particles become closer together and the contact between them increases, due to the reduction in thickness of the diffuse double layer, which leads to an improvement in the mechanical properties of clay (a physical mechanism). Trindade et al.^53^ demonstrated that as a result of the gradual loss of water during thermal treatment, clay minerals undergo a series of changes such as dehydration, dehydroxylation, decomposition and formation of new phases, and vitrification (a chemical mechanism). The majority of water loss occurs during the first and second processes, specifically, the dehydration and dehydroxylation processes. The dehydration process occurs at temperatures between 100 and 200 °C. During this process, only the adsorbed, pore, and interlayer water are removed without any changes in the crystal structure of the clay minerals, thus it does not cause thermal crack growth. Therefore, the improvement in mechanical properties of the mudstone in this study at temperatures of 250 and 500 °C is caused by dehydration process. On the other hand, in the dehydroxylation process that occurs at temperatures between 500 and 1000 °C, hydroxyl ions leave the crystal structure of the clay minerals leading to the growth of thermal cracks which can be seen in Fig. 12 after thermal treatment at temperatures of 600 and 700 °C. Zhang et al.^1^ concluded that the overall structure of the mudstone undergoes phase change at a temperature of about 600 °C, therefore, the mechanical properties of mudstone suddenly change after thermal treatment under this temperature. After this temperature, a chemical reaction of structure occurs, some illite is produced and kaolinite disappears, and finally, the bearing capacity of the mudstone decreases. For temperatures less than 400 °C, no micro-cracks were developed and most pre-existing cracks are intergranular cracks with good closure due to thermal expansion. Micro-cracks were dramatically developed at a temperature between 600 and 800 °C, abundant transgranular, intergranular and intragranular cracks forming a connected network of fractures were observed under 800 °C. In addition, Trindade et al.^53^ concluded the following mineralogical transformation in calcite and dolomite rich clay at temperature between 300 and 1100 °C, K-feldspar and gothics disappeared at 300 °C, kaolinite disappeared at 500 °C and hematite appeared at 500 °C in calcite and 800 °C in dolomite. During dihydroxylation process at T between 500 and 1000 °C, plagioclase, anatase and illite, disappeared at 700, 800 and 900 to 1000 °C, respectively. While quartz disappeared at temperature > 1100 °C.

### Recommendation for future works

This research was conducted after thermal treatment at a temperature range between RT and 500 °C, which is less than the temperature limit of the dihydroxylation process, and under low lateral pressures (0.0 to 4.0 MPa). For future works, we recommend investigating the shear behaviour of intact mudstone during the dehydration and dihydroxylation processes and under high values of lateral pressure. In addition, we recommend using a scanning electron microscope (SEM) and X-ray diffraction (XRD) to investigate the development of thermal microcracks and mineralogical transformations in clay minerals after thermal treatment.

## Conclusions

This study conducted a comprehensive analysis of the impact of thermal treatment and lateral pressure on the shear strength characteristics of intact mudstone by using a Short Core in compression test (SCC). Two methods were adopted to achieve the goal of the current research, numerical and experimental methods. In the numerical method, a numerical code was developed based on the displacement discontinuity method to simulate the SCC test. Shear and maximum stress fields were obtained over the entire SCC samples and compared with experimental observations after the test. The study revealed that when samples were subjected to uniaxial and lateral stress states at various temperatures (RT, 250 °C, and 500 °C), the fractures observed in SCC samples were shear fractures. This was concluded based on their alignment with the vertical shear stress bridge that connects the inner tips of the two horizontal notches, as opposed to being inclined as per the maximum principal stress bridge. By increasing lateral pressure, the impact of tensile stress is reduced and the growth of shear fractures is promoted, and the resulting shear fractures become straighter and more healed.

The experimental work in this study concluded that in comparison with other rock types such as granite and sandstone shown in the literature, shear properties of intact mudstone that include mode II fracture toughness (K_IIC_), peak shear strength (τ_P_), peak friction angle (φ_P_) and the cohesion (C_P_) have only one positive trend with temperature increase up to 500 °C. This is because, until 500 °C, no thermal fractures were developed in the mudstone, the contact between particles increased due to thermal expansion, and clay minerals were dried during the dehydration process that can occur at a temperature between 100 and 200 °C. It is worth mentioning that some thermal fractures were observed after thermal treatment at 600 °C, and a network of these fractures was observed at 700 °C. In addition, the experimental method concluded that the peak fraction angle is less affected by thermal treatment than the cohesion, by increasing T from RT to 500 °C, φ_P_ improved by about 4.9%, while the C_P_ improved by about 47.7%. The bilinear Mohr–Coulomb failure criterion can be used to model the peak shear strength behaviour of intact mudstone before and after thermal treatment.

## Data Availability

The datasets used and/or analysed during the current study available from the corresponding author on reasonable request.
